# Supplementary material for: Anesthetics fragment hippocampal network activity, alter spine dynamics, and affect memory consolidation
Source: PLoS Biol. 2021 Apr 1;19(4):e3001146. doi: 10.1371/journal.pbio.3001146 (PMC8016109; doi:10.1371/journal.pbio.3001146)
Supplement: Attachment — Submitted filename: Hanganu_Wiegert_PLoSBiol.docx [file pbio.3001146.s013.docx]

The authors of the current study investigated and compared the effects of three general anesthetics, isoflurane (Iso), medetomidine/midazolam/fentanyl (MMF), and ketamine/xylazine (Keta/Xyl), on neuronal activity of the mouse hippocampus and memory consolidation at the behavioral level. The neuronal activity was investigated at the local field potential, single unit electrophysiology level, and using chronic two-photon microscopy to monitor calcium transients in large ensembles of neurons and related structural dendritic spine dynamics. The main conclusion is that all three anesthetics reduced neuronal firing rates, decorrelated pairwise neuronal activity, and altered spine dynamics though with distinct signatures. In particular, Iso anesthesia resembled most closely the neuronal activity during wakefulness, was followed by the fastest network recovery, and, contrary to the other two anesthetics, did not impair consolidation of the memory encoded prior to its administration. The authors conclude that the three anesthetics are distinguished in their main effects on hippocampal activity, with implications for their future selection as anesthetics of election for animal research and clinical settings. Overall, the experiments are well designed, the data support the conclusions, the data analysis is comprehensive and appropriate, and the findings are novel (and somewhat in contrast with previous results from the neocortex) and interesting for the broad readership of the journal. In addition, the results have wide implications, particularly when considering that invasive animal research cannot avoid the use of general anesthetics, most of which are comprehensively examined in this study. I only have minor comments that are aimed at improving the overall impact of the study.

1. There are a number of statements for which citations are given, but facts are omitted. The authors should carefully proofread the manuscript and made the facts directly available to the reader (rather than asking the reader to consult the citation). I only list a couple of examples:

- line 71: “…relaying information about external and internal states, respectively”. It is not clear what the authors refer to as internal and external nor whether the CA3 and EC areas convey that type of information to CA1 and how. The authors should elaborate these ideas in the Introduction

- lines 141-144: the authors should clarify whether the wake-up cocktail refers only to MMF anesthesia or they think it may also impact Iso anesthesia

- line 70: it would help to mention stratum lacunosum-moleculare as the target for EC3 input to CA1

2. The same animals were administered with all three anesthetics. It is not clear, nor discussed, the implications of previous anesthesia and of its type on the following ones when it comes to hippocampal network activity. The authors should try to compare the network effects of first-time anesthesia with those of the second-time and more generally with the history of multiple anesthetics used. This would likely add relevance and rigor to the study.

3. There is a body of studies that used wake-up cocktails to study neuronal activity (e.g., intracellular recording of CA1 pyramidal cells in freely-moving rats) and other that used NMDA blockers at subanesthetic doses to study stability or replay of place cell activity in the hippocampus. The authors should connect the results of their current study to some of the findings reported by the other aforementioned studies and comment on the potential implications of using the mentioned protocols on hippocampal activity.

4. The violin plots are used extensively throughout the manuscript. They are good at illustrating data with large ranges, but sometimes are difficult to read. Whenever possible, I would suggest converting those plots to more standard bar graphs with overlaying dot-plots or box-and-whiskers.

5. Some of the two-photon images (Fig. 3B and 4A) appear rotated and re-aligned compared to their counterparts. The authors should explain if the field movement was restricted to the 2-D field or additional 3-D alignment was needed and also why the images under MMF anesthesia appear so different compared with the other conditions.
