# Supplementary material for: Anesthetics fragment hippocampal network activity, alter spine dynamics, and affect memory consolidation
Source: PLoS Biol. 2021 Apr 1;19(4):e3001146. doi: 10.1371/journal.pbio.3001146 (PMC8016109; doi:10.1371/journal.pbio.3001146)
Supplement: Attachment — Submitted filename: response_to_reviewers.docx [file pbio.3001146.s014.docx]

**Response to reviewers**

**Information on all changes applied to figures can be found at the end of the document**

**Reviewer #1:**

This is a very detailed analysis of the effect of three general anesthetics on hippocmapal functions (isoflurane, medetomidine/midazolam/fentanyl and ketamine/xylazine). Authors use multisite recordings of LFP and units, multicellular and spine calcium imaging and behavioral tasks to examine their effects longitudinally in head-fixed mice (except for behav tasks). They found different effects from mild (iso) to disturning (keta/x) on indices of neuronal firing (LFP and unit), correlational firing (calcium imaging, units), spine turnoever (calcium imaging) and episodic memory consolidation (watermaze). They conclude different anesthetics affect hippocampal network dynamics and function differently.

While the issue is not novel, the study is quite compelling in terms of the different preparations and the longitudinal design used to test the effect of the same anesthetics in all animals. The topic is of interest given prevalence of post-operative cognitive decline. I have however several comments regarding the analysis and concerns regarding the reach of conclusions.

We are delighted that the reviewer finds our study compelling and of general interest. Below, please find our response to the criticism. We hope that our additional data and analyses convince the reviewer of the suitability of our manuscript for publication in PloS Biology.

Major:

1- The main conclusion that keta/x works worse than iso and MMF may be quite naive. Iso is a volatile anesthetics and therefore it is not surprising recovery is quite straitforward. MMF can be quickly antagonized as well. This different recovery trends of course have different impact on post-anesthetics cognitive performance. Also, the effect is not only neural, but systemic, affecting the entire organism. So, it is a bit unclear how the different pieces of the analysis can related one each other in a more 'causative' way (ie. whether epidosic memory deffects can be explained by changes in LFP, units, spines...). This is my major concern regarding the reach of conclusions. Actually, in their behav experiments authors directly declare there are differences between groups in terms of the recovery time (page 17, line 539). To my eyes that preclude any possibility to compare cognitive effects and their conclusions on this block are missleading.

We agree with the reviewer that it is important to distinguish between specific effects of the anesthetic and general effects associated with the longevity of the anesthesia or the recovery from it.

To ask whether the impaired performance in the episodic memory task in the water maze experiment was due to a longer period of unconsciousness under Keta/Xyl vs. Iso, or whether the effect was specific to the anesthetic itself, we anesthetized mice with Iso for 1h, 2h or 4h after reversal learning. Unlike for Keta/Xyl and MMF, none of the extended durations of anesthesia with Iso affected memory performance on the next day, indicating that it is not the duration of unconsciousness but rather the type of anesthetic that explains the impaired memory consolidation. The new experiments are presented in Fig. S11B,C and we updated the main text accordingly (lines 655-659 and 838-848).

We further asked how hippocampal activity recovers during the hours following anesthesia, using sequential calcium imaging in CA1 during 6 hours after anesthesia onset. This additional analysis allowed us to directly follow network dynamics during the recovery phase, further demonstrating that Keta/Xyl, but even more MMF (despite antagonization) have long-lasting effects on coordinated neuronal activity. However, the network disturbances are distinct from Iso. This information is now presented in figures 4 (panels D,E) and S6 (panel E) and in a new section of the manuscript (lines 369-388). Together with the new analysis of behavior, this demonstrates that long-lasting alterations of network dynamics alone do not necessarily disrupt memory consolidation. While long-term application of Iso had no effect, the lasting alterations specifically imposed by Keta/Xyl and MMF disrupted memory consolidation.

Finally, we would like to point out that we already considered long-lasting systemic effects of Keta/Xyl in the original manuscript and we also discuss the discrepancy between reversal of physiological vs. hippocampal network dynamics after MMF antagonization (lines 694-698).

2-More specifically, I have some additional comments and concerns that require clarification. First, where are LFP recorded in the dorsal hippocampus? There are laminar variabilities along the structure and I guess authors have homogenized their analysis to specific strata (i.e. SLM?). If so, this should be clarified. Importantly, by collecting LFP signal from only one strata you may miss effects ocurring at other layers, especially if the action of the anethetics differs in terms of excitatory or inhibitiory pathways, for instance. Later in their analysis of dendritic spines authors aim to evaluate different layers but the reader is left with the impression that laminar differences were not appropiately controlled. I recommend authors more consistently including results per lamina in their analysis (i.e. SO, SR, SLM). Here too, having access to spatial LFP signals allow to estimate current-source densities (CSD) which provide more specific information regarding the main input pathways than LFP

This is an important point that we considered in our analysis. To avoid ambiguity due to the laminar organization of the hippocampus, we analyzed the LFP recorded at the recording site in the pyramidal layer of CA1. We apologize for not having pointed this out more clearly. We specify this now in the Methods section (lines 1077-1078).

However, although not the focus of our study, we agree that analyzing the effects of anesthesia in different strata of the hippocampus could help to better understand how activity is locally altered in CA1. We therefore followed the suggestion of the reviewer and compared spine turnover, spine survival and spine density independently for stratum oriens, stratum radiatum and stratum lacunosum moleculare. This analysis is provided in supplemental figure 10 D.

As further suggested, we also analyzed whether there was a layer-specificity of the anesthesia effect on SUA firing rate. Despite some apparent differences between layers, hippocampal layer did not reach statistical significance as a main effect. This new analysis is now presented in figure S2C.

It would certainly be interesting to follow layer-specific effects of anesthesia in CA1 in even more detail. However, this is not the focus of this study and would go beyond the scope of this manuscript.

3-I am unclear how authors have collected information from slow activities given their amplifiers are band-pass filtering signals and operating in AC-coupled. Most of the power spectrum <0.1Hz is confusing to me and does not feel real (directly measured), but the result of some extrapolation. This should be clarified and addressed.

We agree with the reviewer that, in light of the filtering carried out by the amplifier, it is not appropriate to include computations on LFP properties on frequencies below 0.1 Hz. We addressed this by limiting our LFP power (figure 1D and S1C) and PPC analysis (figure 2F) to frequencies above 0.1 Hz. The figures are now corrected. Our new analysis did not affect the conclusions or general findings with respect to the effects of anesthetics.

4- Using the 1/f spectral trend to evaluate E/I ratio is not standard. Authors stand on the Gao et al. 2017 paper but this measurement is not widely accepted. Actually, the 1/f trend more likely reflect the properties of the tissue-electrode interface with the amplifier dominating responses (i.e. one part of 1/f is the electric noise). Biological deviation from that noise reflect physiolopgical activity, and so the measure is circular. I recommend authors using other types of estimation (like interneuron/pyramidal cell rate, etc...)

We agree with the reviewer that using the 1/f decay exponent to measure excitation/inhibition balance is an indirect measure. However, methods based on the firing rate of pyramidal/inhibitory interneurons are also limited as they ignore the different time constant and amplitude of excitatory/inhibitory post-synaptic potentials, and therefore only partially reflect the balance between excitatory and inhibitory synaptic currents. Further, as several interneuron classes are not identifiable by electrophysiological markers alone (i.e. a combination of halfwidth/firing rate/spike waveform asymmetry, etc.), such a measure would require labelling (opto-tagging) of all interneurons, which would be beyond the scope of this manuscript. Despite these caveats, we followed the reviewer’s suggestion and sorted units into fast- and regular-spiking (FS and RS, respectively) ones. When comparing the reduction in firing rate between FS and RS units, we see a tendency (p = 0.058) that goes in the same direction of our findings using the 1/f decay exponent (i.e. a stronger reduction for RS than FS units, see reviewer fig. 1). However, due to the above-mentioned caveats, decided to not include this analysis in the manuscript.

We still believe that the 1/f decay exponent is a valid approach to measure excitation/inhibition balance. The paper by Gao et al., has been cited over 100 times in less than three years, and the theory that it proposes (that the 1/f decay exponent tracks E/I balance) has been validated by experimental (Trakoshis et al., 2020) and theoretical studies (Lombardi, Herrmann, & de Arcangelis, 2017; Trakoshis et al., 2020). Further, the 1/f decay exponent has been used in several papers that are relevant to the arguments that we put forward in our manuscript. In humans, it has shown to track anesthesia administration (Colombo et al., 2019; Lendner et al., 2020), sleep phases (Lendner et al., 2020) and aging (Voytek et al., 2015), in a manner that is consistent with what is the known effect of these conditions on excitation/inhibition balance. To the extent of our knowledge, the method proposed by Gao et al., is the most accepted non-invasive measure of large-scale excitation/inhibition balance. We now point this out more clearly in the revised manuscript (lines 136-140).

**Reviewer figure 1: Modulation of fast spiking and regular spiking units during anesthesia.** Violin plots quantifying the modulation of spike rate in fast spiking vs. regular spiking units during anesthesia compared to wakefulness. n = 4 mice.

5- PPC (Vinck et al.) analysis on Fig.2. depends on the firing rate. Because there are differences in the FR between groups I recommend authors reinforcing this part with an additional measure (i.e. their STTC used later in the ms).

PPC was specifically developed to circumvent the problem that traditional SUA-LFP phase locking measures (such as the PLV) depend on the firing rate. PPC does not (Vinck, van Wingerden, Womelsdorf, Fries, & Pennartz, 2010). STTC is also firing-rate independent, but it is a measure of correlations between spike trains, not a measure of SUA-LFP phase locking. Therefore, we do not see need to change this aspect of our analysis.

6- For the cluster analysis and results it is unclear whether effects depend on one animal or are consistent across mice (Fig.6). A more consistent reporting per animal should be added as suplementary information here.

Indeed, excluding the fact that cluster analysis might depend on a single animal is important. More generally, we agree that it is important to not ignore the (eventual) nested nature of statistical units. However, the statistical methods that we used (linear mixed-effect models) do take in consideration this possibility (animal was used as a fixed effect). We can therefore exclude that the reported effects depend on a single mouse.

To illustrate to the reviewer that the effects seen in our cluster analyses are consistent across animals, we plotted the median values of each animal below together with the violin plots showing the distribution of all individual recordings. Note that the effects in individual animals match the global effects.

**Reviewer figure 2: Fragmented calcium activity in CA1 during anesthesia is consistent between animals.** Violin plots quantifying the number of principal component analysis (PCA) clusters (top left), the power-law slope of the variance explained by the first 100 components (top right), the number of tSNE clusters (lower left), and the number of clusters obtained by affinity propagation (bottom right) during wakefulness or anesthesia, as indicated. Violin plots as in figure 6. In addition, median values are plotted for individual animals. Note that effects in individual animals are consistent.

7- The effects described here may also depend on the age of the animal, gender and invidivual variability of cognitive trends prior to anesthesia (although those can be homogenized). While this is commented in the ms, the reader is left with the impression that there are other critical axes than the one examined here.

We apologize for not pointing out more clearly the measures taken to avoid systematic effects of other variables than the anesthetics. We balanced sexes in all experiments and pseudorandomized the order of treatments between animals.

- Mice of both sexes were used. This is indicated in the Methods section.
- In all experiments, where different anesthetics were applied to the same animals, the order of anesthetics was pseudo-randomized, to avoid systematic effects. This is already indicated in the Methods section.
  - For electrophysiology, see lines 920-921 and Fig. 1A
  - For 2-photon calcium imaging see lines 972-973 and Fig. 3A
  - For 2-photon spine imaging see lines 1022-1023. In this case, we provide all information on the sequence of imaging sessions and the provisions taken to avoid age-related bias in the supplemental figure S10A.
- To avoid the confound of cognitive trends in the water maze experiments, we assigned animals to 4 groups displaying balanced behavioral performance before the reversal learning protocol was started. We now provide additional information how groups were assigned in the Methods section (lines 1067-1068).

**Reviewer #2:**

This manuscript describes the effects of general anesthetics on hippocampal activity, spine turnover, and episodic memory. The authors found that isoflurane (Iso), medetomidine/midazolam/fentanyl (MMF), and ketamine/xylazine (Keta/Xyl) distinctly affected network activity measured by local field potentials, spiking activity, calcium events, spike dynamics in the hippocampal CA1. In addition, they showed that the anesthetics distinctly induced retrograde memory impairments measured in the Morris water maze. I think the analysis were well done, and this manuscript would be a useful catalogue of effects of general anesthetics for the future animal research and clinical settings. However, when thinking about the relationship between hippocampal activity, spine turnover, and episodic memory, the manuscript does not make a strong link between them or shed new light on a major problem in the field, and this I feel is a main shortcoming of the manuscript for PLoS Biology.

We thank the reviewer for the positive and constructive feedback on our manuscript. Indeed, the points raised below would significantly improve the general relevance of the manuscripts. We followed the reviewer’s suggestions and performed electrical recordings as well as calcium imaging during natural sleep.

To enhance the manuscript, I would like to suggest the followings:

1) The manuscript compared the hippocampal activity during waking conscious state and unconscious states under general anesthetics control, and all the anesthetics examined showed distinct activity compared with awake. This is fine, but I believe that more important and intriguing comparison would be between hippocampal activity during natural sleep and under anesthesia, since both periods are unconscious states but only the latter is associated with transient amnesia. In addition, is the neuronal activity under the control of general anesthetics more similar to that in non-REM sleep, which is unconscious state, than that in REM sleep, which is conscious state?

We agree with the reviewer that a comparison between anesthesia and natural sleep would be an important addition to the general interpretability of our data and the relevance of the paper. As suggested, we recorded LFP, SUA and calcium activity in the hippocampus of mice transiting between periods of wakefulness and sleep. Using established electrophysiological features, we categorized the different sleep states and wakefulness. Further, using this dataset as ground truth, we trained a machine-learning algorithm to predict the different behavioral states on eye tracking and pupillometry data alone (Yuzgec, Prsa, Zimmermann, & Huber, 2018), which we then used to predict the behavioral state for the calcium imaging dataset (from which inferring whether the animal is sleeping/awake is more challenging). This procedure is described in a new supplemental figure (Fig. S9) the analysis of all our sleep recordings is shown in a new main figure (Fig. 7).

We further give ample space in the manuscript to the sleep/anesthesia comparison (lines 500-543). In summary, sleep more closely resembled wakefulness than any anesthetic state. Among the different anesthetic conditions, isoflurane is the most comparable to sleep.

2) Firing rates of individual hippocampal pyramidal neurons span at least 3 orders, and neuronal activities of high- and low- firing rate neurons are distinctly affected by natural sleep (Miyawaki and Diba, Current Biology, 2019). Comparison between effects of general anesthetics on high- and low firing rate neurons would give us hints regarding similarity and difference between natural sleep and under anesthesia.

This is an excellent suggestion. To address the point, we developed an analytical strategy to investigate whether neurons are differentially modulated by the different anesthetics and sleep according to their previous firing profile. Unlike sleep, only few neurons increased their activity during anesthesia, while most of them were strongly suppressed. However, consistent with NREM sleep (Watson, Levenstein, Greene, Gelinas, & Buzsaki, 2016), we find an inverse correlation between the awake firing rate (SUA) / calcium transients rate and the reduction induced by all three anesthetics (figs. S2B and S6D). Consistent with previous results (Watson et al., 2016), also in our sleep data we found that NREM sleep differentially affected the firing rate of high and low firing rate neurons, showing a weak negative correlation between initial firing rate and firing rate during sleep (figure 7G).

**Reviewer #3:**

The authors of the current study investigated and compared the effects of three general anesthetics, isoflurane (Iso), medetomidine/midazolam/fentanyl (MMF), and ketamine/xylazine (Keta/Xyl), on neuronal activity of the mouse hippocampus and memory consolidation at the behavioral level. The neuronal activity was investigated at the local field potential, single unit electrophysiology level, and using chronic two-photon microscopy to monitor calcium transients in large ensembles of neurons and related structural dendritic spine dynamics. The main conclusion is that all three anesthetics reduced neuronal firing rates, decorrelated pairwise neuronal activity, and altered spine dynamics though with distinct signatures. In particular, Iso anesthesia resembled most closely the neuronal activity during wakefulness, was followed by the fastest network recovery, and, contrary to the other two anesthetics, did not impair consolidation of the memory encoded prior to its administration. The authors conclude that the three anesthetics are distinguished in their main effects on hippocampal activity, with implications for their future selection as anesthetics of election for animal research and clinical settings. Overall, the experiments are well designed, the data support the conclusions, the data analysis is comprehensive and appropriate, and the findings are novel (and somewhat in contrast with previous results from the neocortex) and interesting for the broad readership of the journal. In addition, the results have wide implications, particularly when considering that invasive animal research cannot avoid the use of general anesthetics, most of which are comprehensively examined in this study. I only have minor comments that are aimed at improving the overall impact of the study.

We thank the reviewer for the positive assessment of the manuscript. Please find our detailed point-by-point response below.

1. There are a number of statements for which citations are given, but facts are omitted. The authors should carefully proofread the manuscript and made the facts directly available to the reader (rather than asking the reader to consult the citation). I only list a couple of examples:

- line 71: “…relaying information about external and internal states, respectively”. It is not clear what the authors refer to as internal and external nor whether the CA3 and EC areas convey that type of information to CA1 and how. The authors should elaborate these ideas in the Introduction

We tried to improve the entire manuscript in this regard and specifically also updated the above-mentioned section. We hope that it is clearer now (lines 67-73).

- lines 141-144: the authors should clarify whether the wake-up cocktail refers only to MMF anesthesia or they think it may also impact Iso anesthesia

MMF requires a wake-up cocktail, for Iso it is sufficient to remove the face mask. We changed the relevant section to point out this difference more clearly (lines 147-149).

- line 70: it would help to mention stratum lacunosum-moleculare as the target for EC3 input to CA1

Done (lines 69-70).

2. The same animals were administered with all three anesthetics. It is not clear, nor discussed, the implications of previous anesthesia and of its type on the following ones when it comes to hippocampal network activity. The authors should try to compare the network effects of first-time anesthesia with those of the second-time and more generally with the history of multiple anesthetics used. This would likely add relevance and rigor to the study.

To avoid systematic, time- or treatment-dependent effects, we shuffled treatments in all experiments. The order of anesthetics was pseudo-randomized during the electrical recordings as well as calcium and spine imaging experiments.

We indicate this in the Methods section and in the respective figure panels:

- For electrophysiology, see lines 920-921 and Fig. 1A
- For 2-photon calcium imaging see lines 972-973 and Fig. 3A
- For 2-photon spine imaging see lines 1022-1023. In this case, we provide all information on the sequence of imaging sessions and the provisions taken to avoid age-related bias in the supplemental figure S10A.
- To avoid the confound of cognitive trends in the water maze experiments, we assigned animals to 4 groups displaying balanced behavioral performance before the reversal learning protocol was started. We now provide additional information how groups were assigned in the Methods section (lines 1067-1068).

3. There is a body of studies that used wake-up cocktails to study neuronal activity (e.g., intracellular recording of CA1 pyramidal cells in freely-moving rats) and other that used NMDA blockers at subanesthetic doses to study stability or replay of place cell activity in the hippocampus. The authors should connect the results of their current study to some of the findings reported by the other aforementioned studies and comment on the potential implications of using the mentioned protocols on hippocampal activity.

We thank the reviewer for pointing this out. We include these aspects in our discussion now. A section mentioning potential confounds of using MMF with a wake-up mix for intracellular hippocampal recordings in freely moving animals is added in lines 789-794.

We further added a section discussing the effects of sub-anesthetic doses of ketamine on place cell activity and episodic-like memory formation in lines 844-848.

4. The violin plots are used extensively throughout the manuscript. They are good at illustrating data with large ranges, but sometimes are difficult to read. Whenever possible, I would suggest converting those plots to more standard bar graphs with overlaying dot-plots or box-and-whiskers.

Bar graphs or, more generally, graphical solutions which do not show single data points, are generally not recommended, something that is even explicitly mentioned in the PLOS Biology guidelines (https://journals.plos.org/plosbiology/s/submission-guidelines#loc-style-and-format). When the number of data points was low, we already resorted to a solution that is not too different than what the reviewer proposes: dot-plots (to illustrate single data points) with overlaid box-and-whiskers (which summarize easy-to-read statistical information such as median/quartiles) and a silhouette (the “violin”) displaying the PDF of the variable (for a better sense of the whole distribution).

5. Some of the two-photon images (Fig. 3B and 4A) appear rotated and re-aligned compared to their counterparts. The authors should explain if the field movement was restricted to the 2-D field or additional 3-D alignment was needed and also why the images under MMF anesthesia appear so different compared with the other conditions.

The 2-photon images shown in figs. 3B and 4A are indeed aligned to each other in order to ensure that the same neurons are considered across all conditions. This procedure is explained in detail in the methods section (under “Calcium imaging data”, “Alignment of multiple recordings”) and in the supplemental material (Figs. S5 and S6). The post-hoc alignment was done only in the X-Y dimensions (2-D). Post-hoc Z-alignment was not possible, because only one plane was sampled to achieve sufficient temporal resolution. However, due to the larger spatial extent of the point-spread function in the Z dimension (Zipfel, Williams, & Webb, 2003), image planes could be readily aligned before acquisition.

We did not observe any particular difference in the images acquired under MMF anesthesia. However, in the particular example shown there were only few neurons active under MMF and thus, a lower number of ROIs is plotted over the images.

**Changes applied to figures compared to original submission**

**Main figures:**

- Fig. 1D, G: replaced with plots that cut-off at 0.1 Hz
- Fig. 2F (left): replaced with plot that cuts-off at 0.1 Hz
- Fig. 4D, E: new panels showing calcium imaging in mice before, during and during 6h after anesthesia with all 3 anesthetics)
- Fig. 6: in panel B MMF and Iso conditions were swapped. Also, panel C was swapped with panel B in S8 (statistics were not affected)
- Fig. 7 (new): showing additional analysis of NREM and REM sleep states (LFP, SUA, calcium imaging)
- Fig. 8D (former 7D): corrected x-axis label from “time (days)” to “time (min)”
- Fig. 9C (former 8C): colors updated to match color scheme

**Supplemental figures:**

- Fig. S1C: top left plot replaced with new plot (0.1-0.5 Hz)
- Fig. S2B,C: new panels showing SUA firing during wakefulness vs anesthesia (B) and layer-dependent effect of anesthesia on SUA (C)
- Fig. S6D,E: showing no. of calcium transients during wakefulness vs. anesthesia (D) and principal component analysis of calcium transients during recovery from anesthesia.
- Fig. S8: in panel A MMF and Iso conditions were swapped. Also, panel B was swapped with panel C in fig.6 (see above - statistics were not affected)
- Fig. S9 (new): showing workflow of sleep scoring in LFP and calcium recording experiments.
- Fig. S10D (former S9): new panel showing analysis of basal, oblique and tuft dendrite spine dynamics.
- Fig. S11 (former S10): in panel A colors for MMF and Keta/Xyl groups were swapped. The plots are replaced with plots showing the modulation index ((d4-d3)/(d3+d4)) instead of relative change. New panels B & C with 1,2, 4 h Iso data

**References**

Colombo, M. A., Napolitani, M., Boly, M., Gosseries, O., Casarotto, S., Rosanova, M., . . . Sarasso, S. (2019). The spectral exponent of the resting EEG indexes the presence of consciousness during unresponsiveness induced by propofol, xenon, and ketamine. *NeuroImage, 189*, 631-644. doi:10.1016/j.neuroimage.2019.01.024

Lendner, J. D., Helfrich, R. F., Mander, B. A., Romundstad, L., Lin, J. J., Walker, M. P., . . . Knight, R. T. (2020). An electrophysiological marker of arousal level in humans. *eLife, 9*. doi:10.7554/eLife.55092

Lombardi, F., Herrmann, H. J., & de Arcangelis, L. (2017). Balance of excitation and inhibition determines 1/f power spectrum in neuronal networks. *Chaos, 27*(4), 047402. doi:10.1063/1.4979043

Trakoshis, S., Martinez-Canada, P., Rocchi, F., Canella, C., You, W., Chakrabarti, B., . . . Lombardo, M. V. (2020). Intrinsic excitation-inhibition imbalance affects medial prefrontal cortex differently in autistic men versus women. *eLife, 9*. doi:10.7554/eLife.55684

Vinck, M., van Wingerden, M., Womelsdorf, T., Fries, P., & Pennartz, C. M. (2010). The pairwise phase consistency: a bias-free measure of rhythmic neuronal synchronization. *NeuroImage, 51*(1), 112-122. doi:10.1016/j.neuroimage.2010.01.073

Voytek, B., Kramer, M. A., Case, J., Lepage, K. Q., Tempesta, Z. R., Knight, R. T., & Gazzaley, A. (2015). Age-Related Changes in 1/f Neural Electrophysiological Noise. *J Neurosci, 35*(38), 13257-13265. doi:10.1523/JNEUROSCI.2332-14.2015

Watson, B. O., Levenstein, D., Greene, J. P., Gelinas, J. N., & Buzsaki, G. (2016). Network Homeostasis and State Dynamics of Neocortical Sleep. *Neuron, 90*(4), 839-852. doi:10.1016/j.neuron.2016.03.036

Yuzgec, O., Prsa, M., Zimmermann, R., & Huber, D. (2018). Pupil Size Coupling to Cortical States Protects the Stability of Deep Sleep via Parasympathetic Modulation. *Curr Biol, 28*(3), 392-400 e393. doi:10.1016/j.cub.2017.12.049

Zipfel, W. R., Williams, R. M., & Webb, W. W. (2003). Nonlinear magic: multiphoton microscopy in the biosciences. *Nat Biotechnol, 21*(11), 1369-1377. doi:10.1038/nbt899
