# Supplementary material for: Anesthetics fragment hippocampal network activity, alter spine dynamics, and affect memory consolidation
Source: PLoS Biol. 2021 Apr 1;19(4):e3001146. doi: 10.1371/journal.pbio.3001146 (PMC8016109; doi:10.1371/journal.pbio.3001146)
Supplement: Attachment — Submitted filename: Response to reviewers revision2.docx [file pbio.3001146.s015.docx]

REVIEWERS' COMMENTS:

Reviewer #1:

Authors have addressed my comments with new data and analysis. I found the current version is improved.

We thank the reviewer for the positive feedback.

I have only few more comments regading data analysis and presentation:

- Violin plots depend on a kernel density function. For cases of somehow homogenegous distribution there is no big issue, but this is not the case for uneven distributions such as in Fig.6. Can authors specify the statistical design shown here? Is the number of animals nested? Is there one particular animal influencing more results than another? Is grouping showing this nested variability? Consider reporting mean data per mouse.

The reviewer is right in pointing out that violin plots depend on a kernel density function. For plotting, we use the “violinplot” function of *seaborn* with parameter cut set to 0, so that the pdf of the variable is not inferred beyond the values that are present in the dataset. To limit the influence of the inferred pdf, when possible (i.e. when the number of single data points was lower than ~100), we also plotted single data points.

For figure 6, as for most of the paper, we resorted to linear mixed-effect models to statistically analyze the data. Such methods have the advantage of causing less type I errors compared to approaches that use nested data points as statistical units (e.g. individual neurons in SUA analysis), while not ignoring the intra-animal variance as would occur by averaging over grouping factors (e.g. averaging SUA results over individual mice). Such methods, in essence, estimate the parameters in a hierarchical manner, as specified by the grouping (random effects) factors. For figure 6, “mouse” was used as a random effect. In the related Github repository, under “Stats_Dataset_(R)”, the reader can find the datasets that were used for statistical analysis and the scripts specifying the model that was used. <https://github.com/mchini/Yang_Chini_et_al/tree/master/Stats_Dataset_(R)>

- Same for Fig.7H,K,L. Can these data be log-normalized? Here too, regarding sleep classification (Fig.7A,I), please provide data point distribution between mice.

Following the suggestion of the reviewer, we added the results of sleep classification per animal to supplemental figure 9 (panel E).

As to the log-normalization, fig. 7K is already log-normalized. Log-normalization of 7H would over-emphasize the long tail of small values in the distribution and would not be consistent with our reporting of the spike-time tiling coefficient in other figure panels of the manuscript (e.g. 5E). Likewise, we did not report the log-normalized absolute correlation coefficient throughout the manuscript. Therefore, to be consistent with the remaining figures, we report this factor on a linear scale also in fig. 7L. Moreover, log-normalization does not reveal any differences in the absolute correlation that we might have missed with our conventional analysis.

Reviewer #2:

I believe that all the issues pointed out by the reviewers have been adequately addressed and resolved.

We thank the reviewer again for the useful and constructive criticism. We are glad we could address all concerns to the reviewer’s satisfaction.
